# Outcomes in Children and Adults With Antiglomerular Basement Membrane Disease

**DOI:** 10.1016/j.ekir.2026.106353

**Published:** 2026-02-13

**Authors:** Adriana Suhlrie, Wilbert A.G. van der Meijden, Stanislas Faguer, Federico Alberici, Anisa Idrizi, Vincent Audard, Aude Servais, Olivia Boyer, Claire Dossier, Jörg Radermacher, Chrysanthi Skalioti, Jakub Zieg, Marina Aksenova, Ann Raes, Thomas Renson, Antonio Mastrangelo, Eiske Dorresteijn, Alexandra Terzi, Antonia Bouts, Ana Fernandes Teixeira, Maria Herrero-Goñi, Dieter Haffner, Nele Kanzelmeyer

**Affiliations:** 1 Department of Pediatric Kidney, Liver, Metabolic and Neurological Diseases, Hannover Medical School, Hannover, Germany; 2 Department of Nephrology, Radboud University Medical Center, Nijmegen, The Netherlands; 3 Department of Nephrology and Organ Transplantation, Centre Hospitalier Universitaire de Toulouse, National Reference Centre for Rare Kidney Diseases, Toulouse, France; 4 Nephrology Unit, University of Brescia, ASST Spedali Civili, Brescia, Italy; 5 Nephrology and Renal Transplantation Department, Assistance Publique-Hôpitaux de Paris, Universite Paris Est Créteil, Institut National de la Santé et de la Recherche Médicale U955, Institut Mondor de Recherche Biomédicale, Henri Mondor University Hospital, Créteil, France; 6 Department of Nephrology and Transplantation, Hôpital Necker Enfants Malades, APHP, Institut Imagine, Université Paris Cité, Paris, France; 7 Néphrologie pédiatrique, Hôpital Necker Enfants Malades, Institut Imagine, Université Paris Cité, Paris, France; 8 Department of Pediatric Nephrology, Robert-Debré Hospital, APHP, Paris, France; 9 Johannes Wesling Klinikum Minden, Minden, Germany; 10 Clinic of Nephrology and Renal Transplantation, Medical School, National and Kapodistrian University of Athens, Athens, Greece; 11 Department of Pediatrics, Second Faculty of Medicine, Charles University and Motol University Hospital, Prague, Czech Republic; 12 Y. Veltischev Research and Clinical Institute for Pediatrics and Pediatric Surgery at the Pirogov Russian National Research Medical University, Moscow, Russia; 13 Department of Pediatric Nephrology and Rheumatology, Ghent University Hospital, Ghent University, ERKNET Centre, Ghent, Belgium; 14 Fondazione IRCCS Ca’ Granda Ospedale Maggiore Policlinico, Milan, Italy; 15 Department of Pediatric Nephrology, Sophia Children’s Hospital, Erasmus Medical Center, Rotterdam, Netherlands; 16 Klinik für Kinderheilkunde II, Universitätsklinikum Essen, Essen, Germany; 17 Emma Children’s Hospital, Department of Pediatric Nephrology, Amsterdam UMC, Amsterdam, The Netherlands; 18 Unidade Local de Saúde de Santo António, Porto, Portugal; 19 Department of Pediatric Nephrology, IIS BioBizkaia Health Research Institute, Cruces University Hospital, University of Deusto, University of the Basque Country UPV/EHU, Barakaldo, Bizkaia, Spain

**Keywords:** antiglomerular basement membrane disease, autoantibodies, children, glomerulonephritis, Goodpasture syndrome, outcome

## Abstract

**Introduction:**

Antiglomerular basement membrane (anti-GBM) disease is a rare, small-vessel vasculitis because of antibodies targeting the glomerular and alveolar capillaries, leading to rapidly progressive glomerulonephritis and/or pulmonary hemorrhage. Data on children with anti-GBM are scarce.

**Methods:**

We collected clinical and biochemical data from European pediatric and adult patients diagnosed with anti-GBM disease between 2020 and 2024.

**Results:**

A total of 72 patients (35% children) with anti-GBM disease and with a median follow-up of 18 months were analyzed. Pediatric cases were more often female and had higher estimated glomerular filtration rate (eGFR) at the time of diagnosis (each *P* < 0.01), whereas the percentage of patients requiring dialysis, presence of pulmonary hemorrhage, and immunological findings did not statistically differ between groups. Treatment consisted mainly of daily plasma exchanges (PEXs)and corticosteroids at higher weight-based doses in children (*P* < 0.0001), in combination with cyclophosphamide (CYC) or, preferably in children (*P* < 0.05), with rituximab (RTX) and mycophenolate mofetil (MMF). Final eGFR was higher in children than in adults (*P* < 0.0001), although the frequency of kidney failure did not significantly differ between children (24%) and adults (38%). Adult patients and patients who required dialysis at the time of diagnosis had a 16-fold and 11-fold increased risk of chronic kidney disease (CKD) stage 3 or higher, respectively.

**Conclusion:**

Our study indicates that girls predominate among children with anti-GBM disease and that children have a better outcome in terms of eGFR than adults, which is at least partly because of better eGFR values at diagnosis. The need for dialysis is a strong predictor of outcome, regardless of age.

## Graphical abstract

**Figure:**
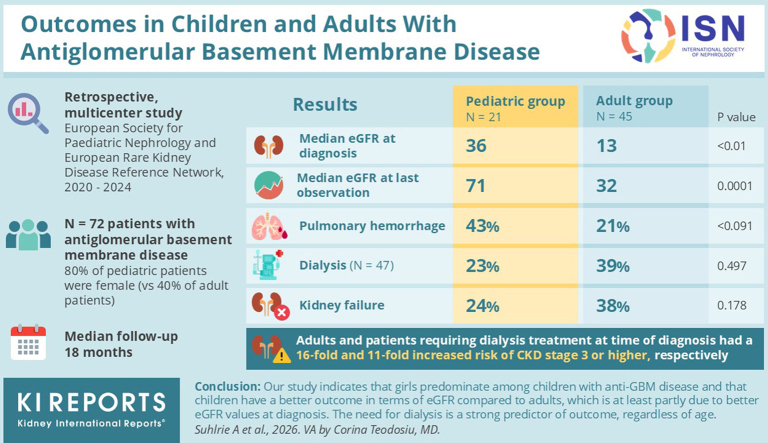

Anti-GBM disease is a rare but rapidly progressive autoimmune disease with an estimated incidence < 1 per million population/yr in European populations.^1^ It is characterized by the presence of autoantibodies against the noncollagenous domain of the α3 chain of type IV collagen and typically manifests as rapidly progressive glomerulonephritis and, in many cases, pulmonary hemorrhage (“Goodpasture syndrome”).^1^ The pathophysiology, clinical course, and therapeutic options (including plasmapheresis, immunosuppression, and supportive measures) in adults are well-described. Oligoanuria and the need for dialysis at the time of diagnosis and the percentage of normal glomeruli in the biopsy are the main predictors of renal outcome in adult patients, which is usually poor with the need for kidney replacement therapy in ≤ 81 % of patients.^2^^,^^3^

This disease is extremely rare in children and adolescents, so only a few small case series and literature reviews based on < 25 patients are available.^4^^,^^5^ Earlier studies describe the clinical course in children as severe, but the available data are limited and heterogeneous.^6^^,^^7^ Furthermore, there are hardly any systematic analyses of treatment response, long-term prognosis, or possible differences compared with adult patient cohorts. The use and patterns of therapeutic plasmapheresis in the pediatric population have also not been sufficiently investigated to date.^8^

Here, we present a retrospective, multicenter study of presentation and outcome in European children and adults diagnosed with anti-GBM disease between 2020 and 2024 from 10 countries. The aim was to identify potential differences between pediatric and adult patients in disease progression and treatment success that are highly relevant for managing this rare but life-threatening disease.

## Methods

### Study Design

An online survey was developed using the platform https://www.soscisurvey.de. Members of the European Society for Paediatric Nephrology and the European Rare Kidney Disease Reference Network who had previously consented to being contacted for data collection received unique participation codes for each patient. Each code enabled submission of anonymized patient data into the survey. The survey was accessible online from July 2024 to May 2025. The corresponding clinicians were contacted where missing or potentially incorrect data (e.g., outliers) was found and asked to complete or verify the data.

Eligible pediatric (aged < 18 years) and adult patients were those with anti-GBM disease, defined as a rapid progressive glomerulonephritis in conjunction with positive anti-GBM antibodies and/or biopsy-proven anti-GBM nephritis which were treated in the last 5 years in the respective centers, that is, between January 2020 and December 2024.^9^ Prospective data entry was possible. The questionnaire included basic information about the reporting physician and center, as well as anonymized patient data collected at diagnosis and at follow-up, that is, at 3, 6, 9, 12, 18, and 24 months, as well as at last visit. Collected variables included age, sex, height, weight, systolic and diastolic blood pressure, serum creatinine, anti-GBM antibody titer, antineutrophil cytoplasmic antibodies (ANCAs) myeloperoxidase and proteinase 3, complete blood cell count, C-reactive protein, protein and albumin-to-creatinine ratios in urine, treatment, kidney biopsy findings, renal (edema, microscopic or macroscopic hematuria, and hypertension), pulmonary (pulmonary hemorrhage and radiological findings), and symptoms. The variable “sex” refers to biological sex (female or male) as documented in the medical records at the time of diagnosis. Information on gender identity was not collected. Therefore, all analyses referring to differences between female and male patients are based on biological sex. Treatment modalities included PEXs with fresh frozen plasma or albumin, immunoadsorption, kidney replacement therapy (dialysis or transplantation), weight-related drug doses, time to treatment initiation after diagnosis, duration of therapy, and number of treatment applications. Hypertension was defined as blood pressure ≥ 95 percentile for age and sex in children and > 140/100 mm Hg in adults.^10^^,^^11^ Kidney biopsy results included the number of cellular and fibrotic crescents. The extent of interstitial fibrosis and/or tubular atrophy (IFTA) was assessed semiquantitatively and graded from 0 to 3 based on the percentage of affected renal parenchyma as follows: grade 0: ≤ 5%, grade 1: 6% to 25%, grade 2: 26% to 50%, and grade 3: > 50% (modified from the Banff Classification).^12^ In addition, adverse events (e.g., leukopenia, infections, and allergic reactions), and pulmonary imaging findings were documented. The study received appropriate ethics committee approval from Hannover Medical School (Ethics vote 11557_BO_K_2024), and from local institutional review boards and was performed in accordance with the Declaration of Helsinki. Written informed consent was obtained from patients or guardians, with consent or assent from patients, as appropriate, including consent to publish the data in anonymized form.

### Clinical, Radiological, and Biochemical Assessments

Blood analyses, histopathological examinations, and radiological assessments were conducted and evaluated at the respective centers in accordance with local protocols and standards. eGFR was calculated according to Pottel *et al.*^13^ Kidney failure at the time of diagnosis was defined as an eGFR < 15 ml/min per 1.73 m^2^ and/or the need for dialysis. At the last observation, kidney function was classified according to the Kidney Disease: Improving Global Outcomes guidelines for CKD stages 1 to 5.^14^ Kidney failure at last observation was defined in patients with CKD stage 5/5D/5T. Creatinine and urea was collected in mg/dl and μmol/l and converted by factors 88.4 and 0.167, respectively. Proteinuria was recorded in g/g, g/mol, and g/m^2^ body surface area (BSA)/d. G/mol was converted by the factor of 8.8 and g/m^2^ body surface area/d by factor 2 into g/g.^15^ Proteinuria was defined as a urine protein-to-creatinine ratio > 0.2g/g. Complete B-cell depletion was defined as CD19^+^ B-cell count < 5 cells/μL for > 9 months.^9^

### Statistical Analysis

Continuous data were expressed as median (interquartile range [IQR]), categorical data were presented as numbers (%). Between-group differences were analyzed using Fisher exact test for categorical variables and using *t* test or Mann-Whitney for numeric variables as appropriate. Longitudinal eGFR changes were analyzed using linear mixed-effects models with time as a fixed effect and subjects as random effects. *Post hoc* comparisons were performed using Tukey's test. Odds ratios and confidence intervals were calculated, as appropriate. Linear and logistic regression analyses were used to evaluate associations between variables. All *P* values were 2-sided and considered significant for values < 0.05. SPSS for Mac, version 31 (IBM Corporation) and GraphPad Prism, version 10.5.0 (GraphPad Software) were used.

## Results

### Patient Characteristics and Clinical Presentation

A total of 72 patients (54% female, 36% children) with anti-GBM disease from 18 centers and a median follow-up of 18 (IQR: 6–44) months were analyzed (Table 1). The median age at diagnosis in children and adults was 14 (IQR 11–16) years and 61 (IQR 47–67) years, respectively (*P* < 0.0001). The proportion of female patients was significantly higher among pediatric than adult patients (20/25 [80%] vs. 19/47 [40%], *P* < 0.01). Twenty-one percent of patients were smokers, and < 1% reported Cannabis intake, which did not statistically differ between groups.

**Table 1:** Characteristics of pediatric and adult patients with anti-GBM disease at the time of diagnosis

Parameters	All patients (*N* = 72)	Pediatric patients (*n* = 25)	Adult patients (*n* = 47)	*P*-value
Age (yrs)	46 (16;63)	14 (11;16)	61 (47;67)	0.0001
Female	39 (54%)	20 (80%)	19 (40%)	0.0025
Smoker	15 (21%)	4 (16%)	11 (24%)	0.366
Cannabis	1 (< 1%)	1 (< 1%)	0 (0%)	0.368
Anti-GBM antibody, IU/l	189 (59;408)	192 (100;294)	184 (43;478)	0.385
ANCA	31 (43%)	7 (28%)	24 (52%)	0.078
MPO (pANCA)	22 (71%)	6 (86%)	16 (67%)	0.426
PR3 (cANCA)	6 (19%)	1 (14%)	5 (21%)	0.414
Not specified	3 (10%)	-	3 (12%)	n.a.
Double positivity	31 (43%)	7 (28%)	24 (52%)	0.078
S-creatinine, μmol/l	445 (151-691)	151 (92;541)	525 (330;740)	0.0007
eGFR, ml/min per 1.73 m^2^	15 (9;36)	36 (11;66)	13 (7;20)	0.0037
Kidney failure^a^	44 (61%)	11 (44%)	33 (70%)	0.043
Dialysis	40 (56%)	11 (46%)	29 (62%)	0.202
U_Prot/Crea_ (g/g)	1.33 (0.46;3.93)	1.52 (0.94;5.42)	0.83 (0.34;2.43)	0.0565
Hematuria (*n* = 62)	61 (98%)	22 (95%)	39 (98%)	0.371
Hypertension (*n* = 68)	32 (47%)	12 (48%)	20 (47%)	> 0.99
Edema (*n* = 68)	20 (30%)	10 (42%)	10 (23%)	0.164

ANCA, antineutrophil cytoplasmic antibodies; eGFR, estimated glomerular filtration rate; GBM, glomerular basement membrane; MPO, myeloperoxidase; n.a., not applicable; PR3, proteinase 3; S, serum; U_Prot/Crea_, urinary protein-to-creatinine ratio.

Data are given as median (interquartile range (IQR)) or number (percentage) as appropriate.

a eGFR < 15 ml/min per 1.73 m^2^ or dialysis.

### Immunology

All but 1 patient had positive anti-GBM antibodies (Table 1). The diagnosis of the anti-GBM antibody-negative patient was based on characteristic kidney biopsy results reviewed by 2 independent renal pathologists. Thirty-one patients (44%) had positive ANCAs, of which 22 (71%) were myeloperoxidase (p-ANCA) and 6 (19%) were proteinase 3 (c-ANCA) positive, and 3 (10%) were not specified. Double positivity (positive anti-GBM and ANCA) was noted in 44% of patients, which tended to be lower in children (28%) than in adults (52%; *P* = 0.078).

### Renal Involvement

**Figure 1:**
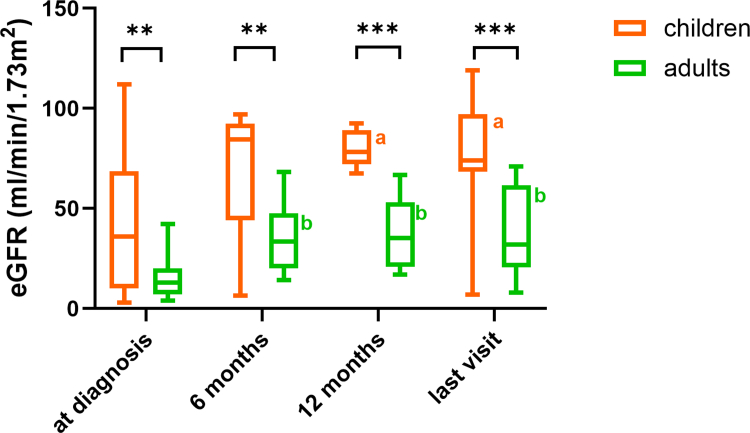
eGFR trajectory in children and adults with antiglomerular basement membrane disease. Median time to last visit: 18 months (interquartile range: 6–44). ^a^ and ^b^ indicate significant differences versus baseline (mixed-effects model analysis, *P* < 0.05). eGFR, estimated glomerular filtration rate.

**Figure 2:**
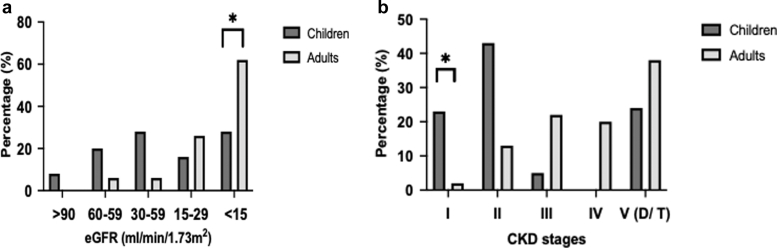
Percentage of pediatric and adult patients with antiglomerular basement membrane disease within (a) the eGFR categories at diagnosis and (b) the CKD stages at the last observation. CKD, chronic kidney disease stage; D, dialysis; eGFR, estimated glomerular filtration rate; T, transplantation.

At the time of diagnosis, pediatric patients had significantly lower median serum creatinine (151 μmol/l vs. 525 μmol/l, *P* < 0.001) and higher eGFR (36 ml/min per 1.73 m^2^ vs. 13 ml/min per 1.73 m^2^, *P* < 0.01) than adult patients (Table 1, Figure 1). A higher proportion of adults had an eGFR < 15 ml/min per 1.73 m^2^ than children (62% vs. 28%, *P* < 0.05), although the proportion of patients receiving dialysis did not statistically differ between groups (62% vs. 46%, *P* = 0.202) (Figure 2a). Proteinuria was detected in 50 patients (86%), with higher median values in pediatric patients than in adult patients (1.52 g/g vs. 0.83 g/g), although this did not reach a level of statistical significance (*P* = 0.056). All but 1 patient presented with hematuria, 40% of them with macroscopic hematuria. Almost half of the patients (47%) were hypertensive, and 30% presented with edema, which did not differ between groups.

### Pulmonary Involvement

Pulmonary hemorrhage was more frequent in children (43%) than in adults (21%), although this did not reach the level of statistical significance (*P* = 0.09). Eighty-five percent of these patients showed critical bleeding, and 6 patients (9%) required mechanical ventilation over a median time of 3.5 days (IQR: 2–10). Pathological findings on imaging were revealed in 68% of cases, notably pleural effusion and ground-glass opacities on X-ray and/or computed tomography (Table 2).

**Table 2:** Extrarenal features and treatment at the time of diagnosis in pediatric and adult patients with anti-GBM disease

Extrarenal features	All patients (*N* = 72)	Pediatric patients (*n* = 25)	Adult patients (*n* = 47)	*P*-value
Pulmonary hemorrhage (*n* = 67)	20 (29%)	10 (43%)	10 (21%)	0.091
Critical bleeding (*n* = 69)	17 (24%)	5 (20%)	12 (26%)	0.772
Mechanical ventilation (*n* = 64)	6 (9%)	3 (14%)	3 (7%)	0.392
Duration of ventilation, d	3.5 (2–10)	4 (3–5)	2 (2–10)	0.600
Pathological findings in x-ray/CT (*n* = 66)	46 (68%)	18 (72%)	28 (64%)	0.602

CT, Computer tomography; GBM, glomerular basement membrane.

Data are given as median (interquartile range) or number (%) as appropriate.

### Kidney Biopsy Results

In 79% of the patients, a kidney biopsy was performed, with pediatric patients being more likely to undergo kidney biopsy (92% vs. 72%, *P* = 0.07). The majority of patients showed both cellular and fibrotic components on renal histology (78% vs. 82%). Pediatric patients showed a higher percentage of cellular (35% vs. 24%, *P* = 0.163) and fibrotic (26% vs. 10%, *P* = 0.685) crescents than adults, although this did not reach a level of statistical significance. The degree of IFTA was generally low and tended to be lower in children than in adults (each *P* > 0.05; Table 3). Only 1 child (6%) and 3 adults (17%) showed grade 2 and 3 IFTA, respectively.

**Table 3:** Kidney biopsy results in children and adults with anti-GBM disease at the time of diagnosis

Biopsy parameters	All patients (*N* = 72)	Pediatric patients (*n* = 25)	Adult patients (*n* = *4*7)	*P*-value
Biopsy performed	56 (79%)	22 (92%)	34 (72%)	0.071
Number of glomeruli	19 (12**–**24)	20 (14–23)	17 (11–25)	0.559
Cellular crescents, %	29 (12–80)	35 (18–90)	24 (9–78)	0.163
Fibrotic crescents, %	10 (2–38)	26 (0.5–58)	10 (2–21)	0.685
IFTA (*n* = 36)		*n* = 18	*n* = 18	
Grade 0 (< 5%)	12 (33%)	8 (44%)	4 (22%)	0.289
Grade 1 (6%–25%)	20 (56%)	9 (50%)	11 (61%)	0.738
Grade 2 (26%–50%)	1 (3%)	0 (0%)	1 (6%)	> 0.999
Grade 3 (> 50%)	3 (8%)	1 (6%)	2 (11%)	> 0.999

GBM, glomerular basement membrane; IFTA, Interstitial fibrosis and/or tubular atrophy of renal parenchyma; grades are semiquantitatively graded based on percentage of affected renal parenchyma.

Data are given as median (interquartile range (IQR) and numbers (percentage) as appropriate.

### Treatment

Sixty-one patients (85%) received PEX, including 10 who underwent double filtration plasmapheresis, and 1 patient who underwent immunoadsorption, which did not significantly differ between groups (Supplementary Table S1). PEXs were mostly performed daily (84%), with 56% and 18% in exchange with albumin and fresh frozen plasma, and 26% in exchange with both. Treatment was started within a median of 1 day (IQR: 1–4) after diagnosis and a median number of 11 PEX cycles (IQR: 6–14) was required for complete clearance of anti-GBM-antibodies, which did not differ between groups. Children were switched to a higher volume per cycle in relation to body weight, than adults (73 ml/kg [IQR: 54–75] vs. 60 ml/kg (IQR: 52–60), *P* = 0.05).

Methylprednisolone pulses were given in 85% of patients with higher body weight–related dosages (17 mg/kg per dose vs. 8 mg/kg per dose, *P* < 0.001) and a higher number of pulses (*P* < 0.01) in children than in adults, followed by oral corticosteroids in 97% of these patients for a median of 6 months (IQR: 4–12) (Supplementary Table S2). The median weight-related glucocorticoid dosages at start of maintenance were higher in children than in adults (1.1 mg/kg vs. 0.9 mg/kg, *P* < 0.0001).

CYC was administered in 79% of the patients, of whom 44% received CYC alone; in 35% of the cases, CYC was combined with RTX and/or MMF. Notably none of the adult patients received MMF. Children received CYC-free treatment more frequently with primary RTX and/or MMF therapy (32% vs. 9%, *P* = 0.018). CYC treatment was started in a median of 2 days after diagnosis, with a median number of 2 (IQR: 4–6) cycles. RTX was given earlier after diagnosis in children than in adults (9 vs. 66 days, *P* = 0.044), whereas the number of RTX administrations and weight-related doses did not differ between groups (Supplementary Table S2). Cotrimoxazol was administered in 74% of patients for a median time of 7 months (IQR: 4–15). One adult patient received avacopan, a complement 5a receptor antagonist, in conjunction with CYC and glucocorticoids.

### Adverse Events

No pediatric patients and 1 adult patient were reported with anaphylaxis to treatment. Leukopenia/infections occurred in 22%/21% of children and 23%/33% of adults (*P* = 0.999 and *P* = 0.375) with the most common infection being pneumonia or respiratory infection (35%). One pediatric patient treated with PEX and CYC, MMF, methylprednisolone pulses, and oral corticosteroids died after 17 months of treatment because of generalized chicken pox infection. B-cell depletion was reported in 11% of patients and did not statistically differ between groups.

### Renal and Pulmonary Outcome

At the last observation with a median follow-up of 18 months (IQR: 6–44), median serum creatinine levels in pediatric and adult patients were significantly lower than at the time of diagnosis, and eGFR was significantly higher (each *P* < 0.0001) (Table 4; Figures 1 and 2). Kidney failure occurred in 35% of patients, with 1 child and 1 adult receiving kidney transplants 1.8 years and 2 years after diagnosis, respectively; and 35% of patients requiring dialysis, which did not statistically differ between groups. In the group of patients who did not require kidney replacement therapy at the last observation, children had lower median serum creatinine (81 vs. 184 μmol/l, *P* < 0.0001) concentrations and higher median eGFR (71 vs. 32 ml/min per 1.73 m^2^, *P* = 0.0001), and more frequently, a normal eGFR value (> 90 ml/min per 1.73 m^2^; 29% vs. 3%, *P* = 0.02) than adults. Proteinuria and hematuria were detected in 51% and 22% of patients, respectively; 51% required antihypertensive therapy. During follow-up, 1 adult patient experienced a relapse in terms of positive anti-GBM antibodies, and 2 patients (1 child and 1 adult) showed a pulmonary relapse with worsening of pulmonary function and/or bleeding. At the last observation, 3% of patients suffered from pulmonary symptoms.

**Table 4:** Outcome in children and adults with anti-GBM disease last observation

Outcome	All patients (*N* = 66)	Pediatric patients (*n* = 21)	Adult patients (*n* = 45)	*P*-value
Time of observation, months	18 (6–44)	17 (7–30)	18 (7–48)	0.625
S-creatinine^a^, μmol/l	124 (87–230)	81 (73–87)	184 (117–279)	< 0.0001
eGFR^a^, ml/min per 1.73 m^2^	48 (26–72)	71 (64–82)	32 (21–53)	0.0001
Kidney failure^b^	23 (35%)	5 (24%)	17 (38%)	0.178
Dialysis (*n* = 47)	16 (35%)	3 (23%)	13 (39%)	0.497
Kidney transplantation	2 (3%)	1 (5%) 1.8 yr after DX	1 (2%) 2 yr after DX	> 0.999
U_Prot/Crea_, g/g	0.20 (0.04–0.51)	0.24 (0.08–0.54)	0.20 (0.05–0.47)	0.675
Hematuria (*n* = 65)	15 (22%)	7 (39%)	8 (18%)	0.1003
Anti-GBM relapse (*n* = 61)	1 (2%)	0 (0%)	1 (2%)	> 0.999
Pulmonary relapse (*n* = 40)	2 (5%)	1 (9%)	1 (2%)	> 0.999

CKD, chronic kidney disease; DX, diagnosis; GBM, glomerular basement membrane; U_Prot/Crea_, urinary protein-to-creatinine ratio.

Data are given as median (interquartile range and numbers (%) as appropriate.

a in patients not on kidney replacement therapy or transplantation (*n* = 48).

b CKD stage 5, 5D (dialysis) or 5T (transplantation).

In the multiple linear regression analysis, chronological age (*P* < 0.00001), anti-GBM antibody titer level (*P* < 0.05), and need of dialysis at time of diagnosis (*P* < 0.05) were independently associated with lower eGFR at last visit (cumulative *R*^*2*^: 0.66). When using categorized age (children vs. adults), age > 18 years (*P* < 0.0001) and need of dialysis at time of diagnosis (*P* < 0.05) were independently associated with lower eGFR at last visit (cumulative *R*^*2*^: 0.642). The multiple logistic regression analysis revealed a 16-fold (95% confidence interval: 3–33, *P* < 0.001) and 11-fold (95% confidence interval: 2–57, *P* < 0.01) likelihood for CKD stage 3 or higher at last observation for adults and patients requiring dialysis treatment at the time of diagnosis. All the other variables, including number of affected crescents and double positivity were no significant correlates of renal outcome.

## Discussion

This study revealed significant differences in presentation and outcome between pediatric and adult patients with anti-GBM disease. Pediatric cases were more often female, and they had better preserved kidney function based on eGFR at the time of diagnosis, whereas the percentage of patients requiring dialysis, presence of pulmonary hemorrhage, and immunological findings did not statistically differ between groups. Children were more likely to receive CYC-free immunosuppression with RTX and/or MMF but received higher weight-based glucocorticoid doses and number of steroid pulses. Children had better renal outcomes in terms of eGFR than adults. Adult patients and patients who required dialysis at the time of diagnosis had a 16-fold and 11-fold increased likelihood of CKD stage 3 or higher at the last observation, respectively.

Previous studies in adult patients with anti-GBM disease showed a slight predominance in males during the first peak incidence in the third decade of life but not in older patients.^16^, ^17^, ^18^ In the present study, 60% of adult patients were female which is in line with a recent analysis in 174 adult anti-GBM patients showing no sex differences.^3^ In contrast, we observed a clear female predominance in the pediatric anti-GBM disease cohort of 80%. The latter could be, at least in part, because of the previously described modulatory effects of estrogens on the production of autoantibodies during adolescent age.^19^

At the time of diagnosis, adults had more severe renal impairment than children, with a significantly lower eGFR despite comparable proteinuria, edema, and hypertension. Kidney biopsies in children tended to show a higher proportion of glomerular cellular crescent formation, the histopathological hallmark of anti-GBM disease, and a lower grade of IFTA than in adults, suggesting more acute inflammatory activity and less (irreversible) chronic changes in children who typically respond better to therapeutic interventions.^3^ This pattern is consistent with the observations of McAdoo and Pusey, who frequently describe advanced kidney involvement, that is, IFTA, in adults at the time of diagnosis.^1^

A salient finding of the present study pertains to pulmonary involvement, wherein the prevalence of pulmonary hemorrhages was found to be higher in children (43%) than in adults (21%), although this difference did not attain statistical significance (*P* = 0.09). A significant proportion of these cases, specifically 85%, were associated with critical hemorrhages. The data presented herein demonstrates that cases of pediatric origin exhibiting severe pulmonary manifestations are not an isolated phenomenon. Although earlier studies indicated a predominance of renal manifestations in children, the present findings suggest that pulmonary involvement in children should not be underestimated. Although limited to single case reports, pulmonary-dominant and even isolated pulmonary presentations have been described in other pediatric patients supporting this notion.^20^^,^^21^

In terms of therapeutic intervention, both pediatric and adult patients rapidly underwent antibody removal by PEX and/or immunoadsorption. This finding aligns with the recommendations outlined in international guidelines and the extant literature on pediatric apheresis.^1^^,^^8^ However, in the present study, a higher weight-related plasma volume per exchange was used in children than in adults, which may have facilitated antibody elimination in the former group. Nevertheless, the number of PEX cycles until anti-GBM antibody titers were no longer detectable, did not differ statistically between the groups and was similar to other reported pediatric cases.^22^, ^23^, ^24^, ^25^ Furthermore, there were observed discrepancies in the administration of immunosuppressive therapy, with children being administered higher weight-related doses of corticosteroids, and higher numbers of steroid pulses. Children more frequently received a CYC-free immunosuppressive with RTX and/or MMF therapy, whereas most adults received CYC. Similar to the Kidney Disease: Improving Global Outcomes guideline for management of ANCA-associated vasculitis, CYC-free immunosuppressive regimens might be preferred by pediatric clinicians to avoid severe side effects such as potential gonadotoxicity affecting pubertal development and future fertility, which is a major consideration during childhood and adolescence but carries less weight in adult patients.^26^

It is noteworthy that despite these differences the number of adverse events, for example, incidence of leukopenia or infections, did not differ between groups. As demonstrated in the preceding case reports and smaller cohorts, RTX has been found to be effective in cases of refractory disease and pediatric cases.^27^ However, there is a lack of systematic data, particularly in pediatric cases, and the small number of patients in the present study does not allow for a sufficiently meaningful statistical analysis of the efficacy of the various immunosuppressive protocols used.^6^

In relation to treatment response, it was demonstrated that there was an improvement in kidney function in both children and adults; however, renal recovery with respect to eGFR was superior in children than in adults. Although 29% of pediatric patients demonstrated the restoration of normal kidney function (eGFR > 90 ml/min per 1.73 m^2^) at last observation, this outcome was observed in a mere 3% of adult patients. This finding is consistent with observations that children have a higher regenerative potential in many kidney diseases and can achieve better functional outcomes with early intervention. In addition, the less pronounced degree of chronic irreversible changes on kidney biopsy in children may have contributed to better renal outcome than in adults, as discussed earlier. Although not assessed in this survey, earlier diagnosis in children, resulting in less acute kidney injury, and classical adult CKD factors, such as diabetes, coronary artery disease, or obesity, may partially have additionally contributed to poorer outcome in adults.^28^

The multivariate analyses of our study confirmed that chronological age, anti-GBM antibody titer levels, and the need for dialysis at diagnosis are independent predictors of long-term renal prognosis. These results are consistent with those observed in large adult cohorts, in which the need for dialysis at diagnosis is a strong negative prognostic factor.^1^ However, it is particularly noteworthy that age had an independent, strong influence on prognosis. Despite the existence of comparable therapeutic interventions, adult patients exhibited a 16-fold elevated risk of developing CKD stage ≥ 3. In contrast to McAdoo *et al.*^1^, double positivity showed no significant impact on renal outcomes in our cohort.

Our study has several limitations. First, the retrospective study design may have biased the results, especially because we cannot be sure that all incident cases and all complications associated with the disease or treatment were reported. Consequently, estimating incidence is beyond the scope of this study. In addition, the time from symptom onset to referral and initial triggers such as infections were not consistently documented. Adult-specific risk factors such as diabetes, coronary artery disease, obesity, were not documented in the survey. Therefore, important determinants of outcome could not be fully assessed.

Second, local biochemical and renal histopathological examinations were performed instead of centralized assessments. In particular, the semiquantitative evaluation of chronic kidney lesions using the adapted Banff criteria is limited by interobserver variability, which may have biased the assessment of chronic damage. In addition, vascular lesions were not assessed. Third, because of the rarity of the disease, our study included a relatively small number of patients, resulting in low statistical power. Fourth, because of the composition of the general population in Europe, predominantly Caucasian children and adults with anti-GBM disease were analyzed. This must be taken into account when translating our results to other populations.

In summary, the findings of this study indicate that females predominate among children with anti-GBM disease, but not in adults; and that pediatric patients derive substantial benefit from early and intensive immunosuppressive therapy, achieving a notably superior renal prognosis in comparison with adult patients, without concomitant elevated rates of adverse effects. Concurrently, the elevated incidence of critical pulmonary hemorrhages in children underscores the imperative for meticulous observation and expeditious intensive care intervention in cases of pulmonary manifestations. These observations suggest that both renal and pulmonary prognosis must be considered separately depending on age. Considering the rarity of the disease, there is a necessity for international registry studies in order to standardize treatment approaches and systematically record age-related differences in prognosis.

## Disclosure

SF received consulting fees from Novartis, Abionyx Pharmacy, CSL-Vifor, and AstraZeneca; as well as fees-fostered Scientific Advisory Board from Novartis, CSL-Vifor, Alexion, and GSK. VA received consulting fees from Addmedica, Vifor, Alnylam, and AstraZeneca outside of the submitted work. OB received speaker fees, and/or consultation fees from Advicenne, Alexion, Alnylam, Biocodex, Chiesi, CSL/Vifor, Novartis, Samsung, Sobi, and Takeda, not pertaining to this work. TR received speaker fees from Alfasigma outside of the submitted work. AM received consulting fees from SOBI, outside of the submitted work. DH received speaker fees, and/or consultation fees from Advicenne, Biologix, Chiesi, Kyowa Kirin, Medison Pharma, Sandoz, and Ultragenyx; and received research grants from Chiesi and Kyowa Kirin. All the other authors declared no competing interests.

## Supplementary Material

Supplementary File (PDF) **Table S1.** Plasma exchange modalities in children and adults with anti-GBM disease. **Table S2.** Immunosuppressive therapy in children and adults with anti-GBM disease.

## Footnotes

Supplementary File (PDF)
**Table S1.** Plasma exchange modalities in children and adults with anti-GBM disease. **Table S2.** Immunosuppressive therapy in children and adults with anti-GBM disease.
