# Supplementary material for: Outcomes in Children and Adults With Antiglomerular Basement Membrane Disease
Source: Kidney Int Rep. 2026 Feb 13;11(5):106353. doi: 10.1016/j.ekir.2026.106353 (PMC13090544; doi:10.1016/j.ekir.2026.106353)
Supplement: Supplementary File (PDF) — Table S1. Plasma exchange modalities in children and adults with anti-GBM disease. Table S2. Immunosuppressive therapy in children and adults with anti-GBM disease. [file mmc1.pdf]

Supplementary material

Table S1: Plasma exchange modalities in children and adults with anti-GBM disease

Therapy	All patients (n=72)	Pediatric patients (n=25)	Adult patients (n=47)	p-value
N	61 (87%)	21 (84%)	40 (87%)	0.712
Daily	51 (84%)	15 (71%)	35 (89%)	0.143
Exchange with FFP (n=34)	6 (18%)	4 (44%)	2 (9%)	0.059
Exchange with albumin	19 (56%)	4 (44%)	15 (65%)	0.069
Exchange with FFP and albumin	9 (26%)	1 (1%)	8 (26%)	0.069
Time from diagnosis to start, days	1 (1;4)	2 (1;6)	1 (0.3;4)	0.164
Number of PEX (n=55)	11 (6;14)	11 (7;16)	11 (6;14)	0.332
Volume/kg body weight per cycle	60 (51;71)	73 (54;75)	60 (52;60)	0.051

Data are given as median (interquartile range (IQR) and numbers (percentage) as appropriate; Abbreviations: FFP, fresh frozen plasma, PEX, Plasma exchange cycles

Table S2: Immunosuppressive therapy in children and adults with anti-GBM disease

Therapy	All patients (n=72)	Pediatric patients (n=25)	Adults patients (n=47)	p-value
**Methylprednisolone pulses**	61 (85%)	22 (88%)	39 (81%)	0.737
Time from diagnosis to start, days	1 (0;2)	2 (1;4)	1 (0;2)	0.2003
Number of pulses	3 (3;3)	3 (3;4)	3 (3;3)	**0.0023**
Dosage, mg/kg	11 (7;15) n=55	17 (14;23)	8 (6;11)	**<0.0001**
Oral glucocortcoids[a]	70 (97%)	25 (100%)	45 (96%)	0.540
Dosage, mg/kg	1 (0.9;1.1)	1.1 (1.0;1.4)	0.9 (0.8;1.0)	**<0.0001**
Duration, months	6 (4;12)	6 (6;11)	7 (3;13.5)	0.916
CYC only	32 (44%)	9 (36%)	23 (49%)	0.329
CYC and RTX and/or MMF	25 (35%)	8 (32%)	17 (36%)	0.798
RTX and/or MMF	12 (17%)	8 (32%)	4 (9%)	**0.0184**
None of the above	3 (4%)	0 (0%)	3 (6%)	0.548
**CYC all**	57 (79%)	17 (68%)	40 (83%)	0.127
Time from diagnosis to start, days	2 (1;8)	5 (2;10)	2 (1;5)	**0.053**
Number of cycles	2 (4;6)	6 (2;6)	3 (2;6)	0.742
Dosage, mg/m^2 BSA per cycle	393 (84;504)	442 (85;509)	396 (85;466)	0.798
**MMF**	10 (42%)	10 (42%)	0	
Time from diagnosis to start, days	31 (11;183)	31 (11;183)		
Dosage, mg/kg per day	33 (31;38)	33 (31;38)		
Duration, months (n=6)	21 (14;24)	21 (14;24)		

**RTX**	33 (46%)	12 (50%)	21 (45%)	0.802
Time from diagnosis to start, days	11 (7;107)	9 (7;13)	66 (9;150)	**0.044**
Number of pulses	4 (2;4)	4 (3;4)	4 (2;4)	0.986
Dosage, mg/m^2 BSA per cycle	370 (311;387)	368 (352;384)	378 (284;521)	0.614
Cotrimoxazole (n=61)	53 (74%)	15 (75%)	38 (86%)	0.276
Duration, months	7 (4;15)	10 (2;24)	7 (4;14)	0.478

Data are given as median (interquartile range (IQR) and numbers (percentage) as appropriate; [a] prednisolone or prednisone; Abbreviations: CYC, cyclophosphamid; RTX, rituximab; MMF, mycophenolate mofetil; BSA, body surface area
